# Anti-Cancer Activity of Phenyl and Pyrid-2-yl 1,3-Substituted Benzo[1,2,4]triazin-7-ones and Stable Free Radical Precursors

**DOI:** 10.3390/molecules23030574

**Published:** 2018-03-03

**Authors:** Lee-Ann J. Keane, Styliana I. Mirallai, Martin Sweeney, Michael P. Carty, Georgia A. Zissimou, Andrey A. Berezin, Panayiotis A. Koutentis, Fawaz Aldabbagh

**Affiliations:** 1 School of Chemistry, National University of Ireland Galway, University Road, H91 TK3 Galway, Ireland; l.keane19@nuigalway.ie (L-A.J.K.); styliana.mirallai@nuigalway.ie (S.I.M.); m.sweeney5@nuigalway.ie (M.S.); 2 Biochemistry, School of Natural Sciences, National University of Ireland Galway, University Road, H91 TK33 Galway, Ireland; michael.carty@nuigalway.ie; 3 Department of Chemistry, University of Cyprus, P.O. Box 20537, Nicosia 1678, Cyprus; zissimou.georgia@ucy.ac.cy (G.A.Z.); berezin@ucy.ac.cy (A.A.B.); koutenti@ucy.ac.cy (P.A.K.); 4 Department of Pharmacy, School of Life Sciences, Pharmacy and Chemistry, Kingston University, Penrhyn Road, Kingston upon Thames, KT1 2EE, UK

**Keywords:** anti-tumour, blatter-type radical, heterocyclic compound, NCI, pleurotin, TEMPO

## Abstract

Cell viability studies for benzo[1,2,4]triazin-7-ones and 1,2,4-benzotriazinyl (Blatter-type) radical precursors are described with comparisons made with 2,2,6,6-tetramethyl-1-piperidinyloxy (TEMPO). All of the stable free radicals were several orders of magnitude less cytotoxic than the benzo[1,2,4]triazin-7-ones. The synthesis and evaluation of two new pyrid-2-yl benzo[1,2,4]triazin-7-ones are described, where altering the 1,3-substitution from phenyl to pyrid-2-yl increased cytotoxicity against most cancer cell lines, as indicated using National Cancer Institute (NCI) one-dose testing. COMPARE analysis of five-dose testing data from the NCI showed very strong correlations to the naturally occurring anti-cancer compound pleurotin. COMPARE is program, which analyzes similarities in cytotoxicity data of compounds, and enables quantitative expression as Pearson correlation coefficients. Compounds were also evaluated using the independent MTT assay, which was compared with SRB assay data generated at the NCI.

## 1. Introduction 

**Figure 1:**
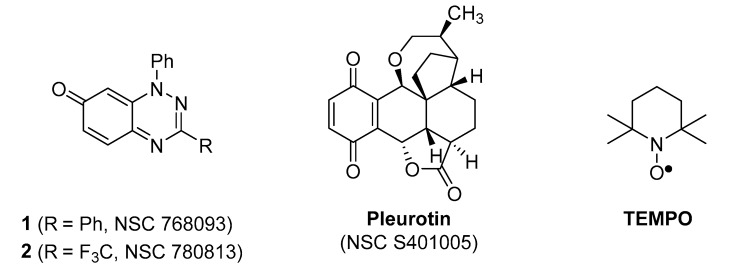
Background: Anti-cancer agents.

**Scheme 1:**
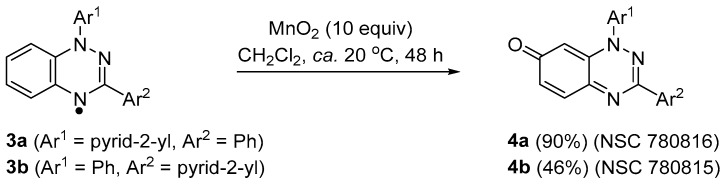
Synthesis of pyrid-2-yl-substituted benzo[1,2,4]triazin-7-ones.

The cytotoxicity of the 3-substituted 1-phenylbenzo[1,2,4]triazin-7-ones **1** and **2** (Figure 1) showed very strong correlation (Pearson correlation coefficients ~0.8) to the naturally occurring antibiotic and anti-cancer agent pleurotin using the National Cancer Institute (NCI) COMPARE analysis. The origin for the anti-cancer activity is likely to be thioredoxin reductase (TrxR) inhibition, with the 3-Ph **1** and 3-F_3_C **2** substituted benzotriazinones exhibiting strong reversible mixed and uncompetitive inhibition [1]. Our anti-cancer studies followed evidence that analogues of scaffold **1** were multi-target inhibitors of Alzheimer’s disease [2]. Benzotriazinones are easily prepared by treating Blatter-type (benzotriazin-4-yl) radicals with manganese dioxide in dichloromethane at room temperature (Scheme 1) [3]. Given that stable free radicals such as 2,2,6,6-tetramethyl-1-piperidinyloxy (TEMPO) have shown cytotoxicity, including against breast (MDA-MB 231) [4] and prostate cancer (DU-145) cell lines [5], in this article we investigate the cytotoxicity of benzotriazin-4-yl radicals **3a** and **3b** and contrast them with the oxidation products **4a** and **4b**. Herein, we report the synthesis and cytotoxicity evaluation of new benzotriazinones, 3-phenyl-1-(pyrid-2-yl)benzo[1,2,4]triazin-7-one (**4a**) and 1-phenyl-3-(pyrid-2-yl)benzo[1,2,4]triazin-7-one (**4b**). Incorporating pyridine is reported to significantly alter cytotoxicity against certain cancer cell lines, hence we made the choice of replacing phenyl in the parent structure **1** with the pyrid-2-yl substituent in **4a** and **4b** [6,7,8].

## 2. Results and Discussion

### 2.1. Synthesis of Pyrid-2-yl-Substituted Benzo[1,2,4]triazin-7-ones

The preparation of benzotriazinyl radicals **3a** and **3b** has previously been reported [9]. New pyridyl-substituted benzotriazinones **4a** and **4b** were prepared from the MnO_2_-mediated oxidation [3] of benzotriazin-4-yls **3a** and **3b** in 90 and 46% yields, respectively (Scheme 1). It should be noted that 1,3-bis(pyrid-2-yl)benzo[1,2,4]triazin-7-one is derived from the MnO_2_-mediated oxidation of 1,3-bis(pyrid-2-yl)-1,4-dihydro-1,2,4-benzotriazine (i.e., the 4-NH, and not the free radical). However, the latter di-substituted pyrid-2-yl derivative was not selected for evaluation at the National Cancer Institute (NCI, see below), and thus is not a subject of the present article.

### 2.2. Development Therapeutic Program (DTP) National Cancer Institute (NCI) 60 Human Tumor Cell Line Screen and COMPARE Analysis

**Figure 2:**
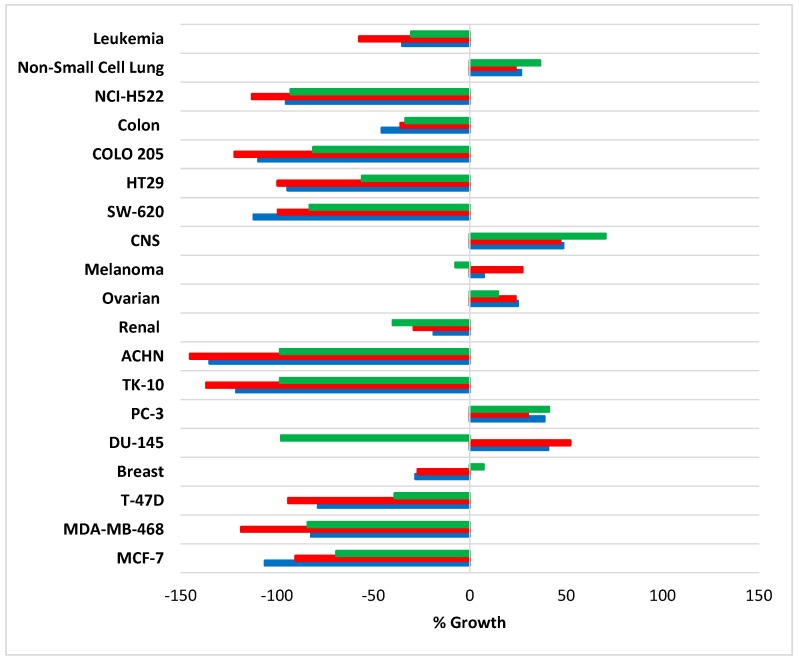
Summary of Development Therapeutic Program (DTP) National Cancer Institute (NCI)-single dose (10 µM) screening results for benzo[1,2,4]triazin-7-ones **1** (green), **4a** (red), and **4b** (blue) expressed as an average percent growth of each cancer cell type or certain cancer cell line relative to untreated cancer cells.

Radicals **3a** and **3b** were not selected by the NCI for cytotoxicity evaluation, although benzotriazinones **4a** and **4b** were used for one-dose (10 µM) screening against the DTP 60 cell line panel. Pyrid-2-yl-substituted compounds **4a** and **4b** exhibited similar variable cytotoxicity profiles, with overall cytotoxicity greater than the 1,3-diphenyl-substituted compound **1** [1] against most of the nine major histological tissue types at the NCI DTP (Figure 2). There was particularly strong growth inhibition exhibited by **4a** and **4b** in certain cancer cell lines, NCI-H522 (non-small cell lung cancer), COLO 205 (colon cancer), HT29 (colon cancer), SW-620 (colon cancer), ACHN (renal cancer), TK-10 (renal cancer), T-47D (breast cancer), MDA-MB-468 (breast cancer), and MCF-7 (breast cancer). Pyridyl-substituted compounds **4a** and **4b**, however, showed little growth inhibition of the DU-145 (prostate cancer) cell line after one-dose testing in comparison to **1**. However, IC_50_ values after NCI five-dose testing (see below) showed that **4b** had comparable cytotoxicity to **1**, with **4a** being over eight times less cytotoxic than **1** against the DU-145 cell line (Table 1). The DU-145 and MCF-7 cell lines were available to us (see below), and the MTT assay was used to obtain independent IC_50_ values.

The NCI selection of **4a** and **4b** for subsequent five-dose testing established key parameters used in the NCI COMPARE algorithm to determine closely matching cytotoxicity profiles [1,10] (complete one- and five-dose data for **4a** and **4b** can be found in the Supplementary Information accompanying this article). The COMPARE analysis facilitated comparisons of cytotoxicity with the NCI’s vast database of over 250,000 synthetic compounds. The degree of similarity between two cytotoxicity profiles is described by the Pearson product-moment correlation coefficient (0 to ±1), with values above ±0.5 considered to be strong. On par with the Pearson correlation coefficient of **1** [1], very strong correlations to pleurotin of 0.84 and 0.73 were obtained for **4a** and **4b**, respectively (Table 2). Altering the 1,3-substitution from Ph to pyrid-2-yl in the benzo[1,2,4]triazin-7-one scaffold did not appear to alter the compound’s mechanism of action, with cytotoxicity profiles closely matching that of the irreversible TrxR inhibitor pleurotin.

### 2.3. Cytotoxicity against DU-145 and MCF-7 Cell Lines Using the MTT Assay

The DU-145 (prostate cancer) and MCF-7 (breast cancer) cell lines, available at the National University of Ireland Galway (NUI Galway), were used to independently determine IC_50_ values. The cytotoxicity of benzotriazin-4-yl radicals **3a** and **3b**, TEMPO, and iminoquinones **4a** and **4b** was determined using the MTT assay. Radicals **3a** and **3b** exhibited very similar cytotoxicity profiles (see Supplementary Information), and were on average 13 and 105 times less cytotoxic towards the DU-145 and MCF-7 cell lines than oxidation products **4a** and **4b** (Table 3). Radicals **3a** and **3b** exhibited an approximate 10-fold greater cytotoxicity towards the prostate cancer compared to the breast cancer cell line. Benzotriazinones **4a** and **4b** gave similar submicromolar IC_50_ values against both cell lines, which were similar in magnitude to that of the previously evaluated compound **1** using the MTT assay [1]. In comparison, TEMPO was found to be relatively non-toxic. This is perhaps not surprising given that high concentrations of TEMPO (of 2.5 mM for 24 h) were required in order to induce a 3.4-fold increase in both early apoptotic cells and late apoptotic/necrotic cells compared with untreated DU-145 cell controls [5]. Literature reports support the requirement for high concentrations (2.5–10 mM) of nitroxide radicals (such as TEMPO) to achieve a therapeutic dose against various breast and prostate cancer cell lines [4,5]. Nevertheless, all radicals were significantly more cytotoxic towards the DU-145 cell line than the MCF-7 cell line.

Using the MTT assay (Table 3), **1**, **4a**, and **4b** exhibited greater cytotoxicity towards DU-145 and MCF-7 cell lines than that shown by the NCI five-dose data (except for **1** against MCF-7 at the NCI Table 1). The discrepancies in IC_50_ values were expected given the longer exposure time of cancer cells to the cytotoxic agent in the MTT assay (72 h versus 48 h at the NCI), as well as fundamental differences in the assays [11,12,13]. The SRB assay used by the NCI measures the amount of dye (sulforhodamine B) bound onto cellular protein, and is reported to be more sensitive than MTT [12,13]. In contrast, MTT relies on the ability of viable cells to reduce the tetrazolium dye MTT (3-(4,5-dimethylthiazol-2-yl)-2,5-diphenyltetrazolium bromide) [11]. 

## 3. Experimental Section

### 3.1. General Procedures

Reactions were protected from atmospheric moisture using CaCl_2_ drying tubes. All volatiles were removed under reduced pressure. All reaction mixtures and column eluents were monitored by TLC (thin layer chromatography) using commercial glass-backed thin layer chromatography (TLC) plates (Merck Kieselgel 60 F_254_, Darmstadt, Germany). The plates were observed under UV light at 254 and 365 nm. The technique of dry flash chromatography was used throughout for all non-TLC scale chromatographic separations using Merck Silica Gel 60 (less than 0.063 mm) (Merck, Darmstadt, Germany) [14]. Melting and decomposition points were determined using either a PolyTherm-A, Wagner & Munz, Kofler-Hotstage Microscope apparatus (Wagner & Munz, Munich, Germany) or a TA Instruments DSC Q1000 (TA Instrument, New Castle, DE, USA) with samples hermetically sealed in aluminum pans under an argon atmosphere, using heating rates of 5 °C/min. Solvents used for recrystallization are indicated after the melting point. UV spectra were obtained using a Shimadzu UV-1601 UV/Vis spectrophotometer (Shimadzu, Kyoto, Japan) and inflections are identified by the abbreviation “inf”. IR spectra were recorded on a Shimadzu FTIR-NIR Prestige-21 spectrometer (Shimadzu, Kyoto, Japan) with a Pike Miracle Ge ATR accessory and strong, medium, and weak peaks are represented by s, m, and w, respectively. ^1^H and ^13^C-NMR spectra were recorded on a Bruker Avance 500 machine (at 500 and 125 MHz, respectively) (Bruker, Billerica, MA, USA). Deuterated solvents were used for homonuclear lock and the signals are referenced to the deuterated solvent peaks. MALDI-TOF MS was conducted on a Bruker BIFLEX III time-of-flight (TOF) mass spectrometer (Bruker, Billerica, MA, USA). 3-Phenyl-1-(pyrid-2-yl)-1,4-dihydrobenzo[*e*][1,2,4]triazin-4-yl (**3a**) and 1-phenyl-3-(pyrid-2-yl)-1,4-dihydrobenzo[*e*][1,2,4]triazin-4-yl (**3b**) were prepared according to literature procedures [9].

### 3.2. Synthesis of Phenyl- and Pyrid-2-yl-Substituted Benzo[1,2,4]triazin-7-ones ***4a*** and ***4b***

*3-Phenyl-1-(pyrid-2-yl)benzo[e]*[1,2,4]*triazin-7(1H)-one* (**4a**). To a stirred solution of 3-phenyl-1-(pyrid-2-yl)-1,4-dihydro-1,2,4-benzotriazin-4-yl (**3a**) (285 mg, 1.0 mmol) in dichloromethane (10 mL), MnO_2_ (869 mg, 10.0 mmol) was added and the reaction mixture was stirred at ca. 20 °C for two days. The reaction mixture was filtered through Celite^®^ (Honeywell Specialty Chemicals Seelze GmbH, Seelze, Germany), rinsed with additional dichloromethane (50 mL), and volatiles were removed in vacuo. The residue was chromatographed on a short pad of silica with *tert*-butyl methyl ether (TBME) to give the title compound **4a** (270 mg, 90%) as purple needles, m.p. (hot-stage): 210.1–213.8 °C (from PhH); m.p. (DSC) onset: 209.9 °C, peak max: 210.5 °C, decomp. onset: 225.8 °C, decomp. peak max: 227.7 °C (from PhH); *R*_f_ 0.58 (TBME, 100%); found: C, 71.87; H, 4.12; N, 18.70. C_18_H_12_N_4_O requires C, 71.99; H, 4.03; N, 18.66%; *λ*_max_(DCM)/nm 295 (logε 4.58), 310 inf (4.46), 339 inf (3.91), 356 inf (3.91), 544 (3.75), 582 (3.70), 635 inf (3.33); ν_max_/cm^−1^ 3073w (aryl C-H), 1624s, 1599s, 1587s, 1570m, 1547s, 1526m, 1497w, 1468m, 1437s, 1398w, 1385w, 1333w, 1310w, 1281w, 1238m, 1192m, 1150w, 1115w, 1103w, 1094w, 1072w, 1028w, 995w, 974w, 905w, 856s, 787s,781m, 760s, 737s; *δ*_H_ (500 MHz, CDCl_3_) 8.63 (1H, d, *J* 3.5 Hz, Ar *H*), 8.26–8.25 (2H, m, Ar *H*), 8.02 (1H, ddd, *J* 8.0, 8.0, 1.5 Hz, Ar *H*), 7.80 (1H, d, *J* 8.0 Hz, Ar *H*), 7.66 (1H, d, *J* 9.5 Hz, Ar *H*), 7.48–7.46 (4H, m, Ar *H*), 7.25 (1H, C*H*, overlap with CDCl_3_), 6.78 (1H, d, *J* 2.0 Hz, C*H*); *δ*_C_ (125 MHz, CDCl_3_) 183.2 (s, C=O), 156.5 (s), 154.6 (s), 150.0 (s), 148.5 (d), 141.5 (d), 139.5 (d), 134.3 (s), 133.7 (s), 132.7 (d), 130.6 (d), 128.8 (d), 126.7 (d), 124.3 (d), 120.0 (d), 99.9 (d); *m/z* (MALDI-TOF) 302 (MH^+^ + 1, 16%), 301 (MH^+^, 100), 300 (M^+^, 21), 299 (M^+^ − 1, 39), 272 (16).

*1-Phenyl-3-(pyrid-2-yl)benzo[e]*[1,2,4]*triazin-7(1H)-one* (**4b**). To a stirred solution of 1-phenyl-3-(pyrid-2-yl)-1,4-dihydrobenzo[*e*][1,2,4]triazin-4-yl (**3b**) (285 mg, 1.0 mmol) in dichloromethane (10 mL), MnO_2_ (896 mg, 10.0 mmol) was added and the reaction mixture was stirred at ca. 20 °C for two days. Upon completion, the reaction mixture was filtered through a short pad of Celite^®^, rinsed with additional dichloromethane (50 mL), and volatiles were removed in vacuo. The residue was chromatographed on a short pad of silica (acetone) to give the title compound **4b** (138 mg, 46%) as purple needles; m.p. (hot-stage): decomp: 199.8–204.0 °C (from PhH); m.p. (DSC) decomp. onset: 205.8 °C, peak max: 210.8 °C (from PhH); *R*_f_ 0.69 (CHCl_3_/MeOH, 80:20); found: C, 71.85; H, 4.13; N, 18.48. C_18_H_12_N_4_O requires C, 71.99; H, 4.03; N, 18.66%; *λ*_max_(DCM)/nm 240 (logε 4.15), 300 (4.46), 310 inf (4.41), 338 inf (4.02), 351 inf (4.05), 374 inf (3.75), 534 (3.64), 572 (3.60), 628 inf (3.24); ν_max_/cm^−1^ 3040w (aryl C-H), 1624m, 1611m, 1589m, 1584m, 1541s, 1537s, 1522m, 1493m, 1477m, 1456m, 1435m, 1395m, 1331m, 1304m, 1233m, 1200m, 1150w, 1117m, 1096w, 1072w, 1045w, 1026w, 995w, 978w, 928w, 907w, 856s, 822m, 814m, 800m, 779s, 764m, 745m; *δ*_H_(500 MHz, CDCl_3_) 8.84 (1H, d, *J* 4.4 Hz, Ar *H*), 8.31 (1H, d, *J* 7.9 Hz, Ar *H*), 7.88–7.83 (2H, m, Ar *H*), 7.65–7.55 (5H, m, Ar *H*), 7.41 (1H, dd, *J* 6.9, 5.2 Hz, Ar *H*), 7.32 (1H, dd, *J* 9.8, 1.9 Hz, C*H*), 6.09 (1H, d, *J* 1.7 Hz, C*H*); *δ*_C_(125 MHz, CDCl_3_) 182.2 (s), 155.3 (s), 151.6 (s), 150.2 (d), 149.8 (s), 142.5 (d), 140.7 (s), 137.0 (d), 136.6 (s), 132.4 (d), 130.3 (d), 130.1 (d), 125.7 (d), 124.7 (d), 122.3 (d), 98.4 (d); *m/z* (MALDI-TOF) 302 (MH^+^ + 1, 13%), 301 (MH^+^, 100), 300 (M^+^, 19), 285 (19), 273 (28), 242 (35).

### 3.3. Cell Culture and Cytotoxicity Evaluation

#### 3.3.1. Materials and Cell Lines

2,2,6,6-Tetramethyl-1-piperidinyloxy (TEMPO, CAS number 2564-83-2) was obtained from Sigma-Aldrich (Darmstadt, Germany). The cytotoxicity evaluation of 1,3-bisphenylbenzo[1,2,4]triazin-7-one **1** was previously reported [1]. The MCF-7 breast cancer cell line and DU-145 prostate cancer cell line were obtained from Dr. Stephen Rea, National University of Ireland Galway (Galway, Ireland).

DU-145 was grown in RPMI-1640 medium and supplemented with 1% 2 Mm L-glutamine, 1% penicillin-streptomycin, and 10% non-heat inactivated fetal bovine serum (FBS). MCF-7 was cultured in Dulbecco’s modified Eagle’s medium (DMEM) containing high glucose (4.5 g/mL) and supplemented with 1% penicillin-streptomycin and 10% heat-inactivated fetal bovine serum (FBS). All cells grew as adherent cultures. Cell culture reagents were obtained from Sigma-Aldrich. Disposable sterile plastic ware was obtained from Sarstedt (Numbrecht, Germany). 

#### 3.3.2. Cytotoxicity Measurements Using the MTT Assay

The MTT colorimetric assay was used to determine cell viability [11]. Cells were added to 96-well plates at a cell density of 1000 cells per well for MCF-7 (200 µL per well) and 2000 cells per well for DU-145 (200 µL per well), and allowed to adhere over 24 h. Compound solutions were added in DMSO (1% *v*/*v* final concentration in well). The control cells were exposed to the same concentration of the vehicle control alone (DMSO). All cells were incubated at 37 °C and 5% CO_2_ (humidified atmosphere) for 72 h. MTT (20 µL, 5 mg/mL solution) was added and the cells were incubated for a further 3 h. The supernatant was then removed using a multi-transfer pipette and DMSO (100 µL) added to dissolve the MTT formazan crystals. The absorbance was determined using a plate reader at 550 nm with a reference at 690 nm. Cell viability is expressed as a percentage of the vehicle-only treated control (DMSO). Dose-response curves were analyzed by non-linear regression analysis and IC_50_ values were determined using GraphPad Prism software, v 8.0 (GraphPad Inc., San Diego, CA, USA). The in vitro activity of the drugs towards all cell lines is expressed as IC_50_ (i.e., concentration required for the reduction of the mean cell viability to 50%).

## 4. Conclusions

All three stable free radicals evaluated were significantly more cytotoxic towards DU-145 than the MCF-7 cell line. Benzotriazin-4-yl radicals **3a** and **3b** were significantly less cytotoxic than their oxidation products, benzo[1,2,4]triazin-7-ones **4a** and **4b**, towards the cancer cell lines evaluated. Pyridyl-substituted benzotriazin-7-ones exhibited submicromolar cytotoxicity using the MTT assay on par with 1,3-bisphenylbenzo[1,2,4]triazin-7-one **1**. The variable DTP-NCI one-dose testing cytotoxicity profiles for **4a** and **4b** led to their selection for five-dose testing. COMPARE analysis demonstrated very strong correlations to pleurotin, despite the overall greater cytotoxicity of the pyrid-2-yl-substituted compounds compared to **1** after one-dose testing.

## Figures and Tables

**Table 1:** NCI IC_50_ data after five-dose testing: DMSO solution of compound (100 µL) was added to plates, which were incubated for 48 h at 37 °C using 5% CO_2_ (humidified atmosphere). Sulforhodamine B (SRB) assay was used to evaluate cytotoxicity.

Compound	IC_50_ DU-145 (µM)	IC_50_ MCF-7 (µM)
**1**	3.388	0.295
**4a**	27.542	3.388
**4b**	5.248	1.348

**Table 2:** NCI Pearson correlation coefficients obtained by COMPARE analysis to pleurotin.

Compound	Pleurotin
**1**	0.841
**4a**	0.842
**4b**	0.730

**Table 3:** Cytotoxicity evaluation (NUI Galway) using the MTT colorimetric assay. IC_50_ values were obtained after the incubation of cells with the test compounds in DMSO for 72 h. IC_50_ for **1** were previously obtained under identical conditions [1].

Compound	IC_50_ DU-145 (µM)	IC_50_ MCF-7 (µM)
**1**	0.230 ± 0.03	0.810 ± 0.08
**TEMPO**	151.400 ± 7.480	>999
**3a**	3.140 ± 0.179	34.740 ± 6.236
**3b**	3.177 ± 0.154	26.415 ± 2.538
**4a**	0.245 ± 0.003	0.303 ± 0.009
**4b**	0.241 ± 0.011	0.277 ± 0.002

## Supplementary Materials

Supplementary materials are available online. Figures S1–S4: ^1^H and ^13^C-NMR Spectra for Compounds **4a** and **4b**, Figure S5–S6: NCI-60 One-Dose Mean Graph Data for Compounds **4a** and **4b**, Figures S7–S8: NCI-60 Five-Dose Mean Graph Data for Compounds **4a** and **4b**, Figures S9–S13: Viability of DU145 and MCF-7 cell lines as determined using the MTT assay for Compounds TEMPO, **3a**, **3b**, **4a** and **4b**.
