# Supplementary material for: Anti-Cancer Activity of Phenyl and Pyrid-2-yl 1,3-Substituted Benzo[1,2,4]triazin-7-ones and Stable Free Radical Precursors
Source: Molecules. 2018 Mar 3;23(3):574. doi: 10.3390/molecules23030574 (PMC6017941; doi:10.3390/molecules23030574)
Supplement: Supplementary file 1 [file molecules-23-00574-s001.pdf]

SUPPLEMENTARY MATERIAL for

Anti-cancer Activity of Phenyl and Pyrid-2-yl 1,3-Substituted Benzo[1,2,4]triazin-7-ones and Stable Free Radical Precursors

Lee-Ann J. Keane, Styliana I. Mirallai, Martin Sweeney, Michael P. Carty, Georgia A. Zissimou, Andrey A. Berezin, Panayiotis A. Koutentis, and Fawaz Aldabbagh*

INDEX

Figures S1-S4 ^{1}H and ^{13}C-NMR Spectra for Compounds **4a** and **4b**

Figure S5-S6 NCI-60 One-Dose Mean Graph Data for Compounds **4a** and **4b**

Figures S7-S8 NCI-60 Five-Dose Mean Graph Data for Compounds **4a** and **4b**

Figures S9-S13 Viability of DU145 and MCF-7 cell lines as determined using the MTT assay for Compounds **TEMPO**, **3a**, **3b**, **4a** and **4b**

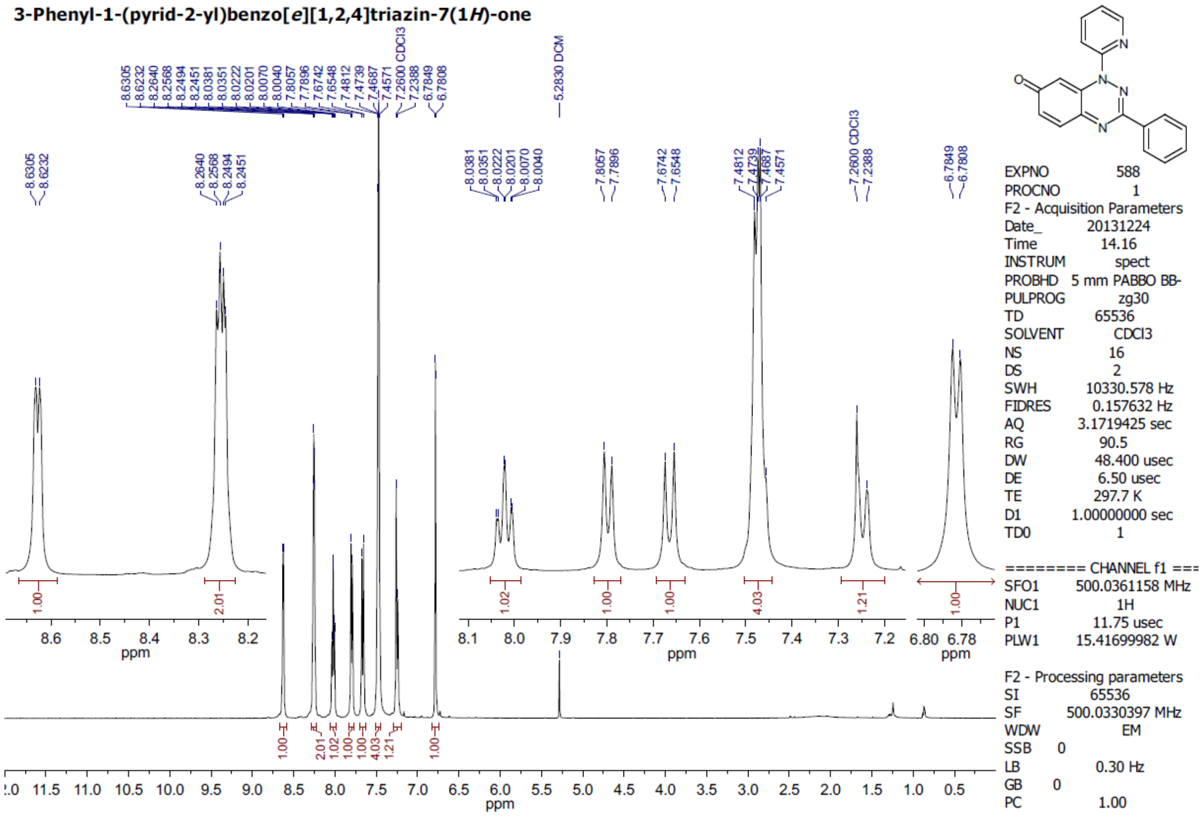

Figure S1. ^{1}H NMR (500 MHz) spectrum in $CDCl_3$ of 3-phenyl-1-(pyrid-2-yl)-benzo[*e*][1,2,4]triazin-7(1*H*)-one (**4a**).

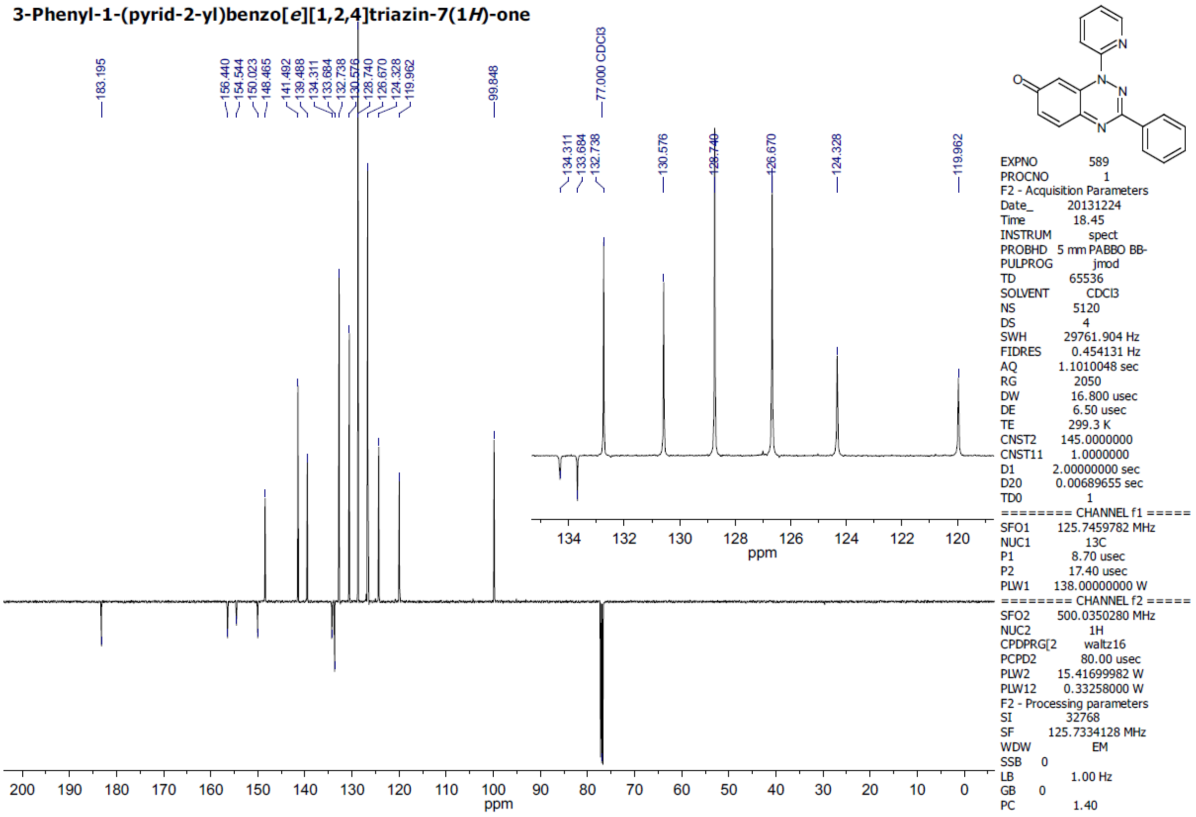

Figure S2. ^{13}C NMR (125 MHz) spectrum in $CDCl_3$ of 3-phenyl-1-(pyrid-2-yl)-benzo[*e*][1,2,4]triazin-7(1*H*)-one (**4a**).

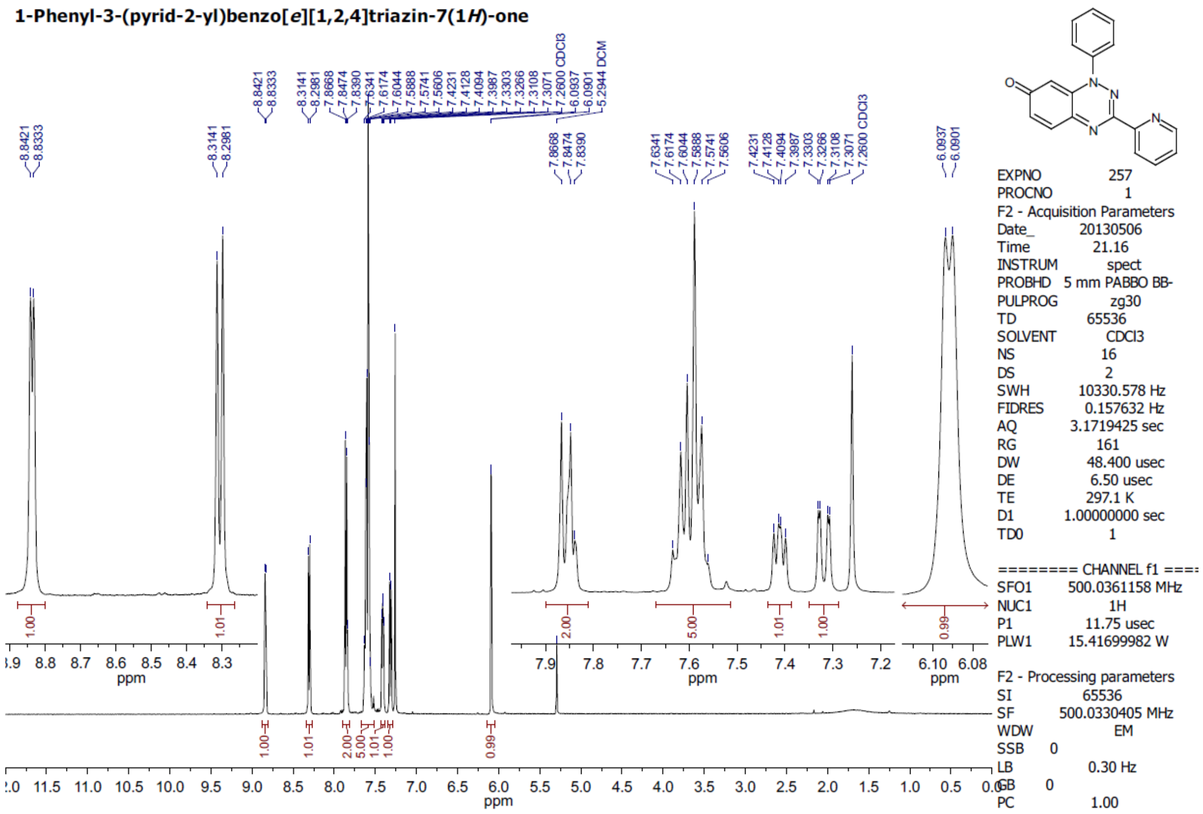

Figure S3. ^{1}H NMR (500 MHz) spectrum in $CDCl_3$ of 1-phenyl-3-(pyrid-2-yl)-benzo[*e*][1,2,4]triazin-7(1*H*)-one (**4b**).

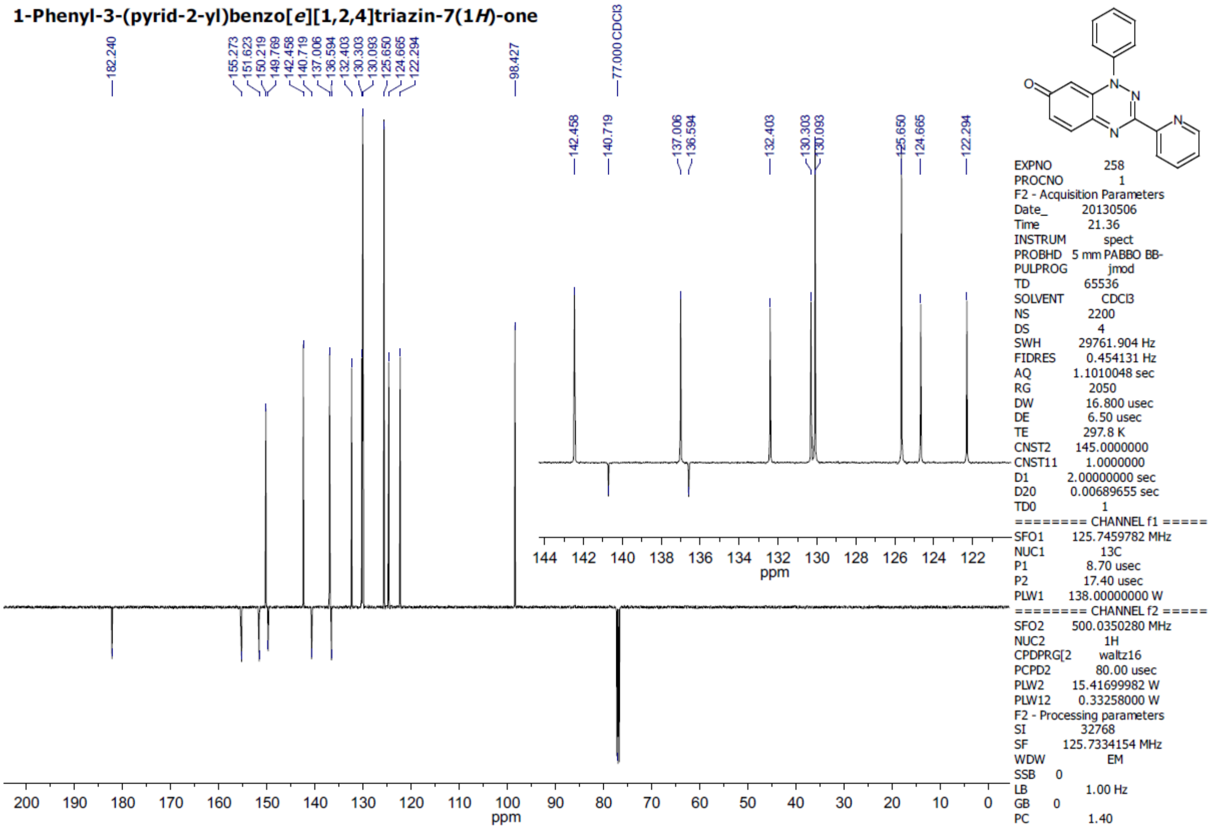

Figure S4. ^{13}C NMR (125 MHz) spectra in $CDCl_3$ of 1-phenyl-3-(pyrid-2-yl)-benzo[*e*][1,2,4]triazin-7(1*H*)-one (**4b**).

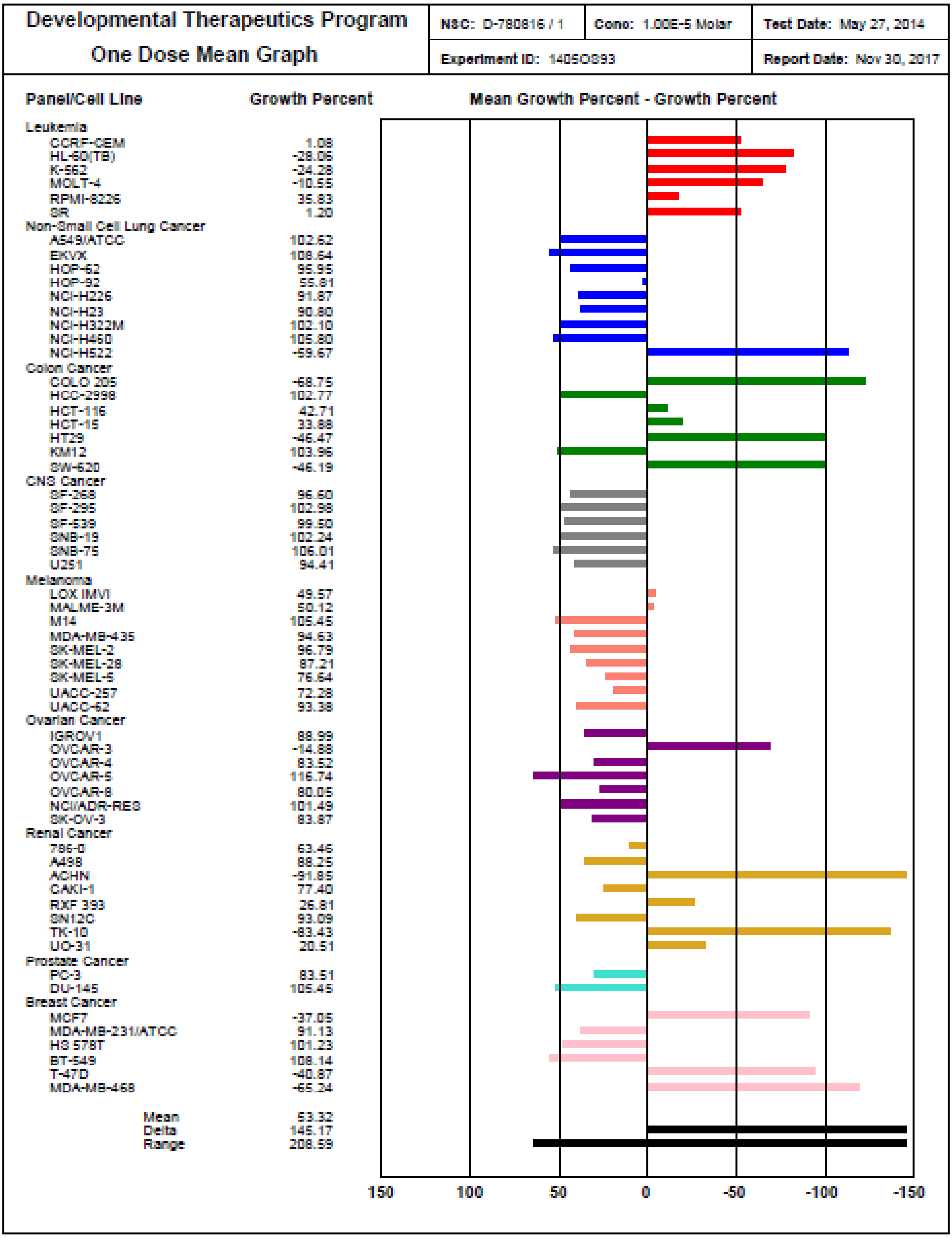

Figure S5. One dose mean graph data for 3-phenyl-1-(pyrid-2-yl)benzo[*e*][1,2,4]triazin-7(1*H*)-one (**4a**) towards the NCI-60 cell line panel.

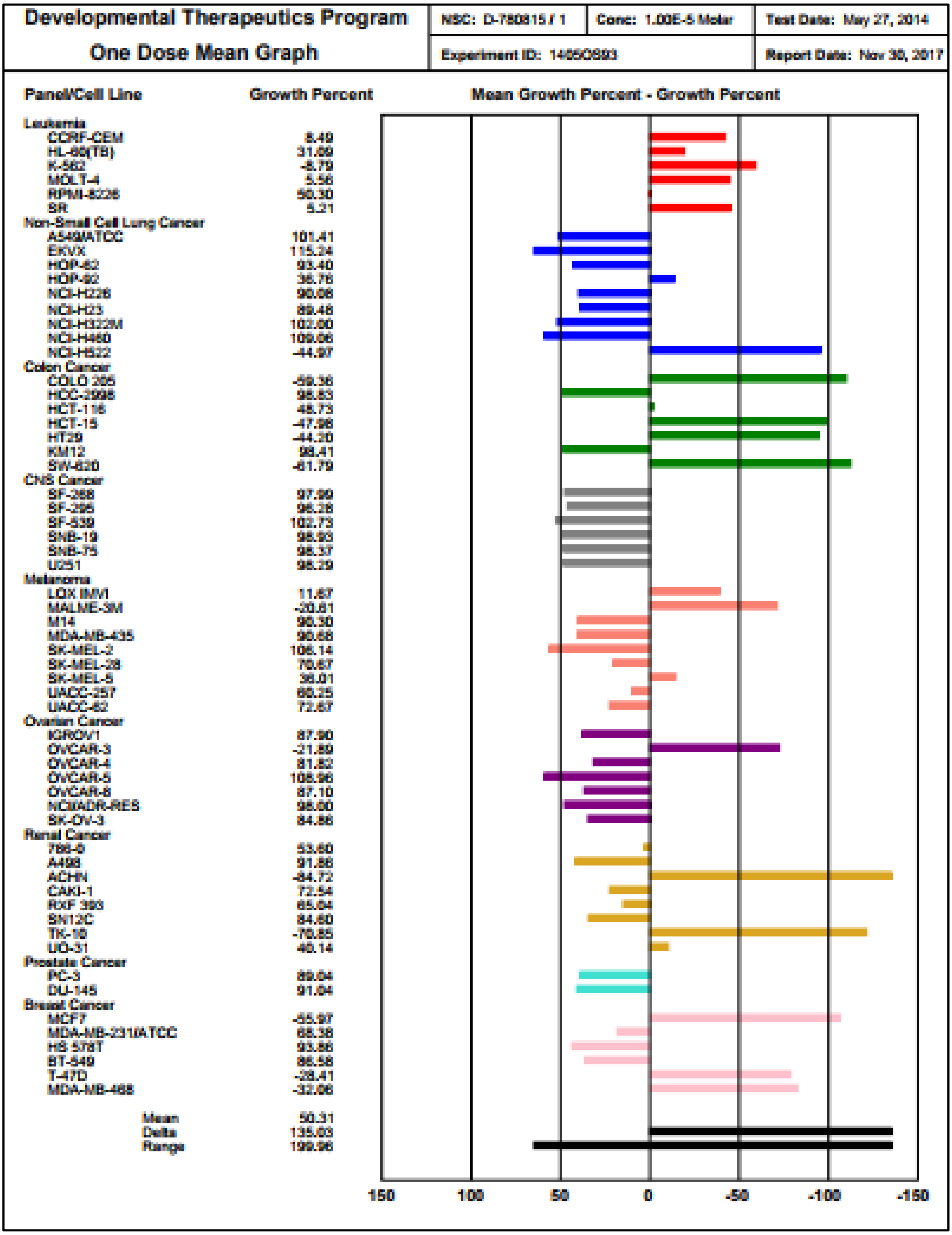

Figure S6. One dose mean graph data for 1-phenyl-3-(pyrid-2-yl)benzo[*e*][1,2,4]triazin-7(1*H*)-one (**4b**) towards the NCI-60 cell line panel.

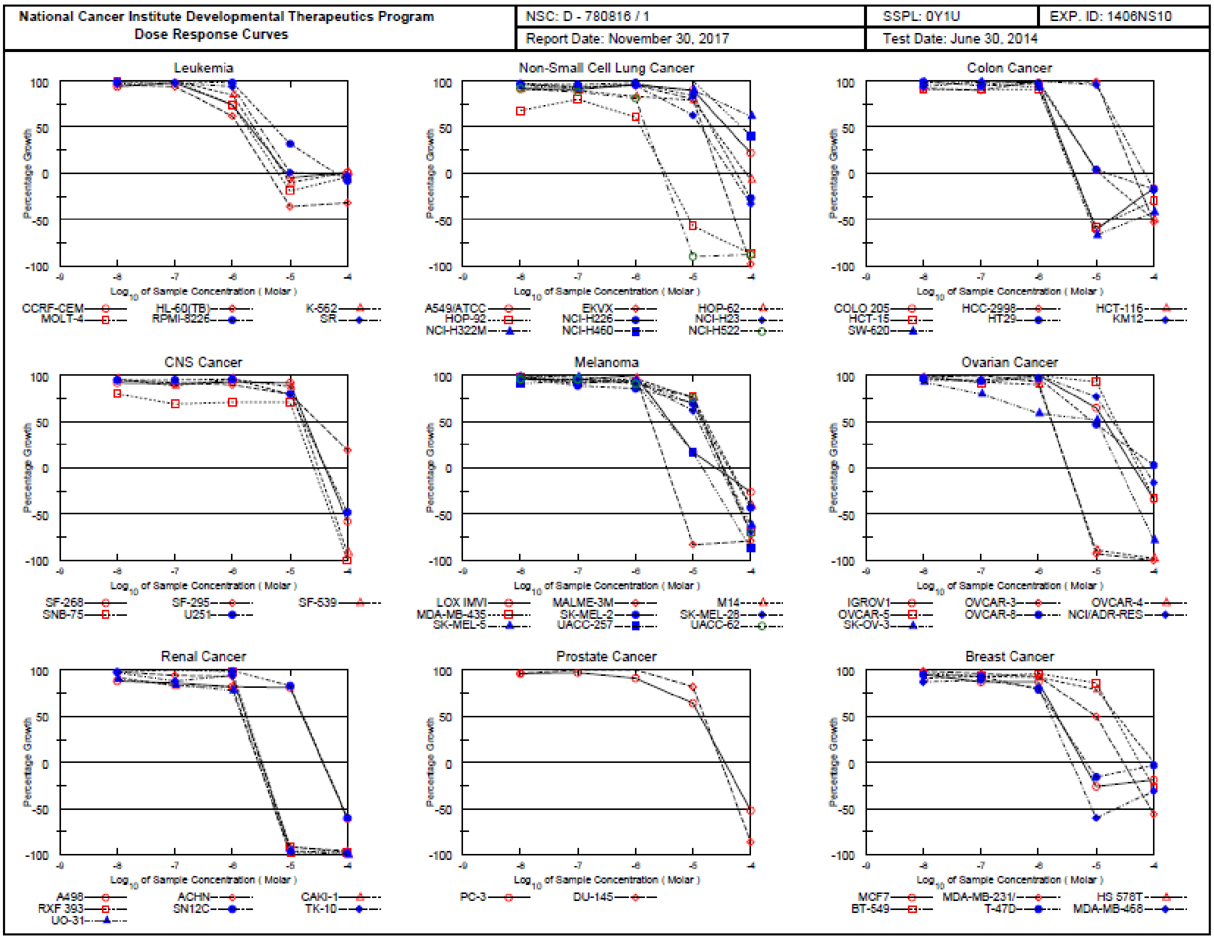

Figure S7. Five dose data for 3-phenyl-1-(pyrid-2-yl)benzo[*e*][1,2,4]triazin-7(1*H*)-one (**4a**) towards the NCI-60 cell line panel.

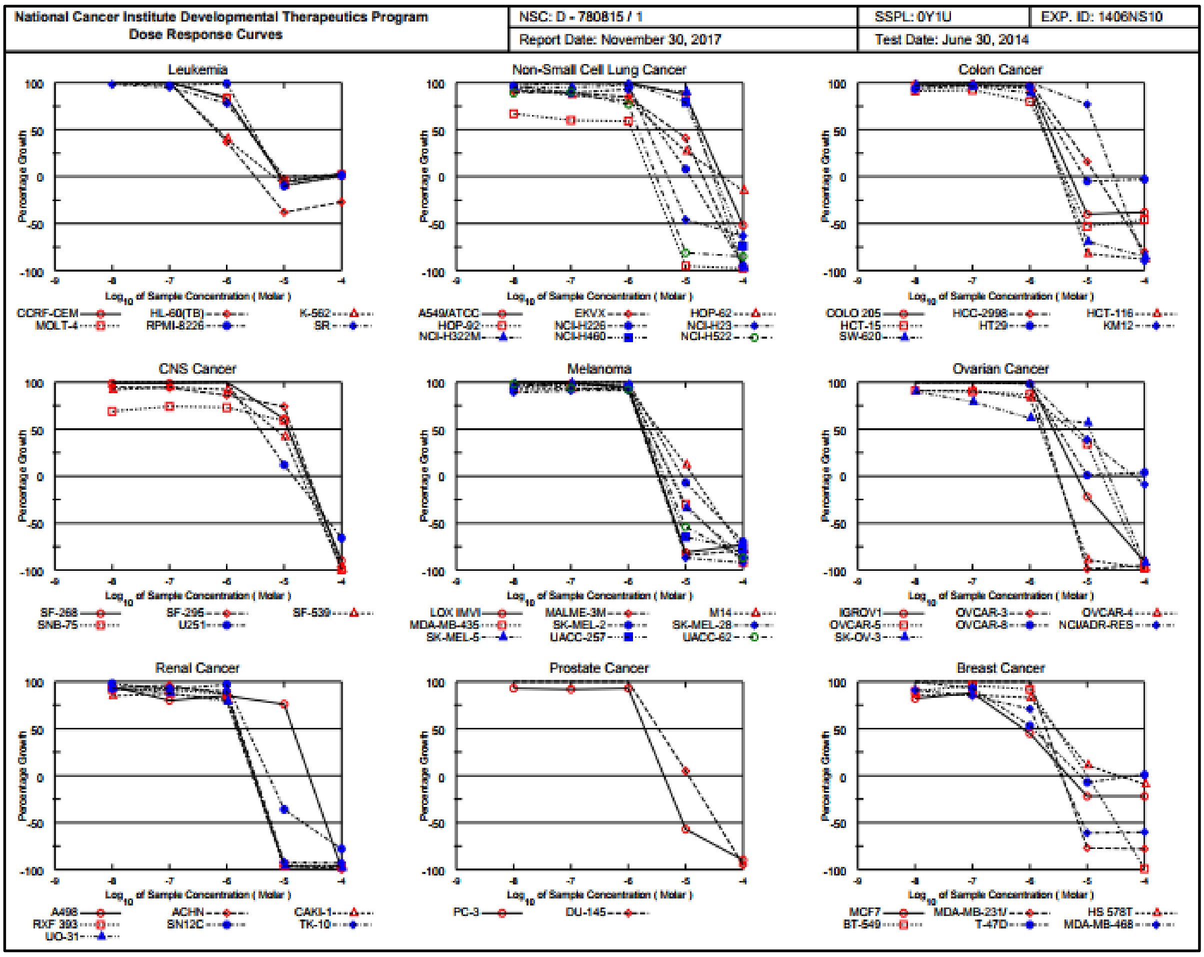

Figure S8. Five dose data for 1-phenyl-3-(pyrid-2-yl)benzo[*e*][1,2,4]triazin-7(1*H*)-one (**4b**) towards the NCI-60 cell line panel.

Cell Viability Graphs

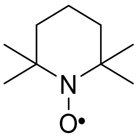

TEMPO

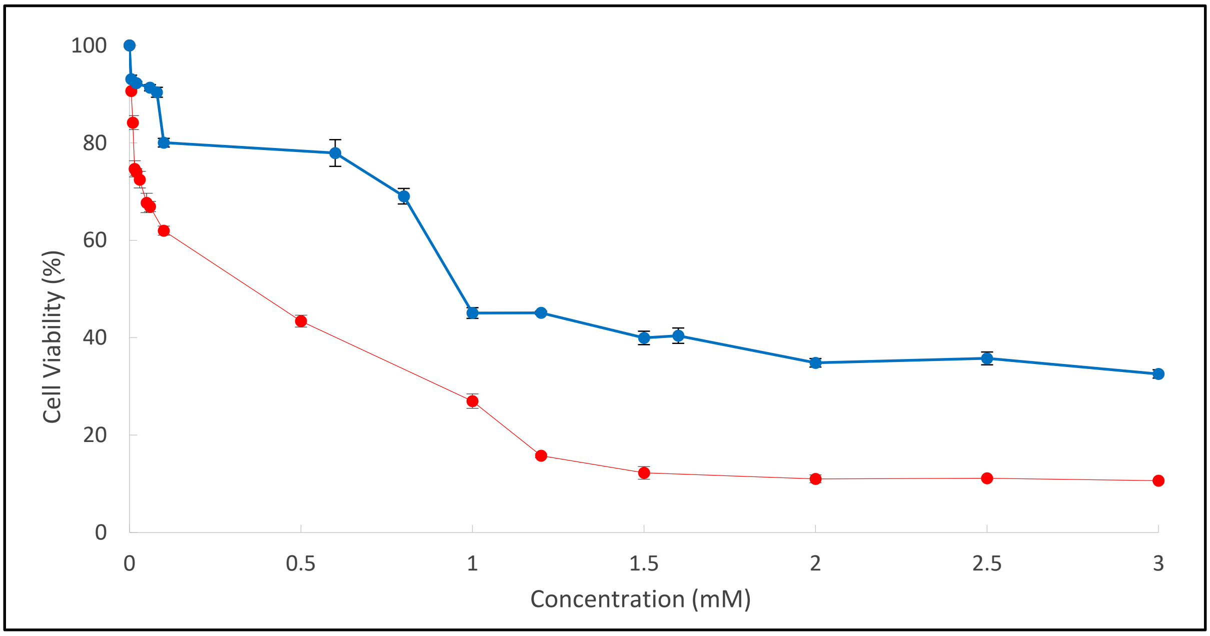

Figure S9. Viability of DU-145 (●) and MCF-7 (●) cell lines determined using MTT assay following treatment with TEMPO under aerobic conditions for 72 h at 37 °C. Each data point is the mean of at least two independent experiments. The lines shown are trend lines.

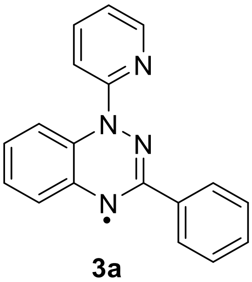

3a

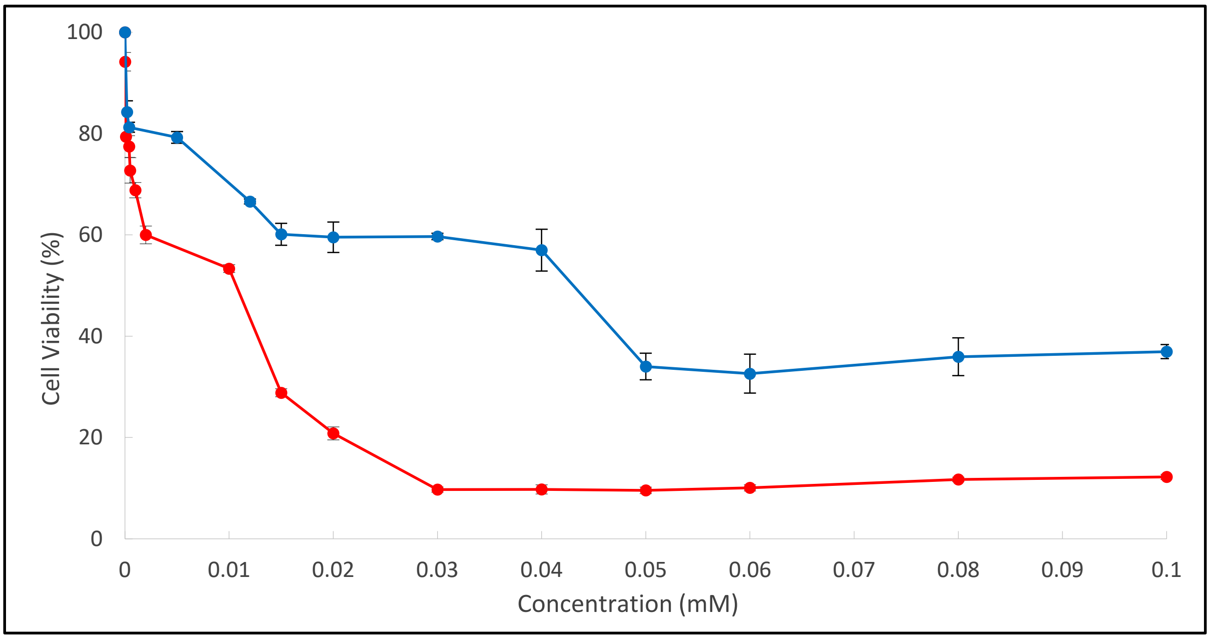

Figure S10. Viability of DU-145 (●) and MCF-7 (●) cell lines determined using MTT assay following treatment with 3-phenyl-1-(pyrid-2-yl)-1,4-dihydrobenzo[*e*][1,2,4]triazin-4-yl (**3a**) under aerobic conditions for 72 h at 37 °C. Each data point is the mean of at least two independent experiments. The lines shown are trend lines.

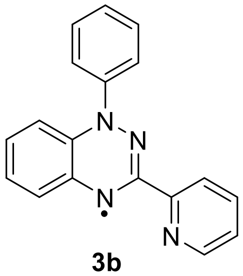

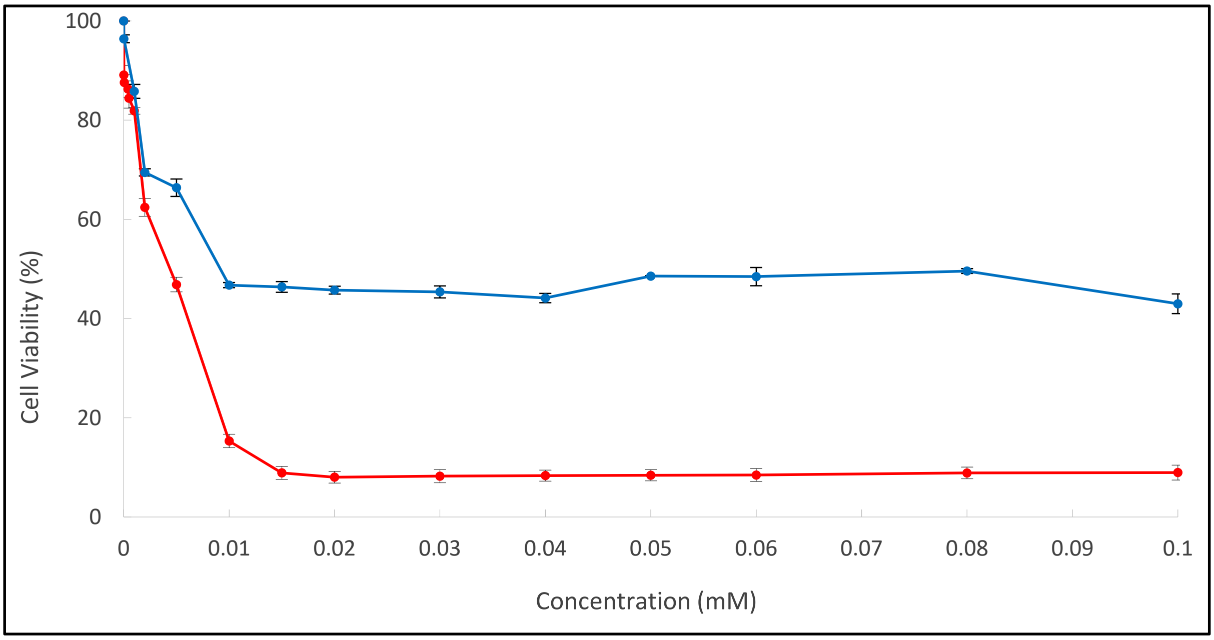

Figure S11. Viability of DU-145 (●) and MCF-7 (●) cell lines determined using MTT assay following treatment with 1-phenyl-3-(pyrid-2-yl)-1,4-dihydrobenzo[*e*][1,2,4]triazin-4-yl (**3b**) under aerobic conditions for 72 h at 37 °C. Each data point is the mean of at least two independent experiments. The lines shown are trend lines.

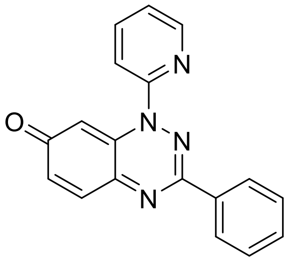

4a

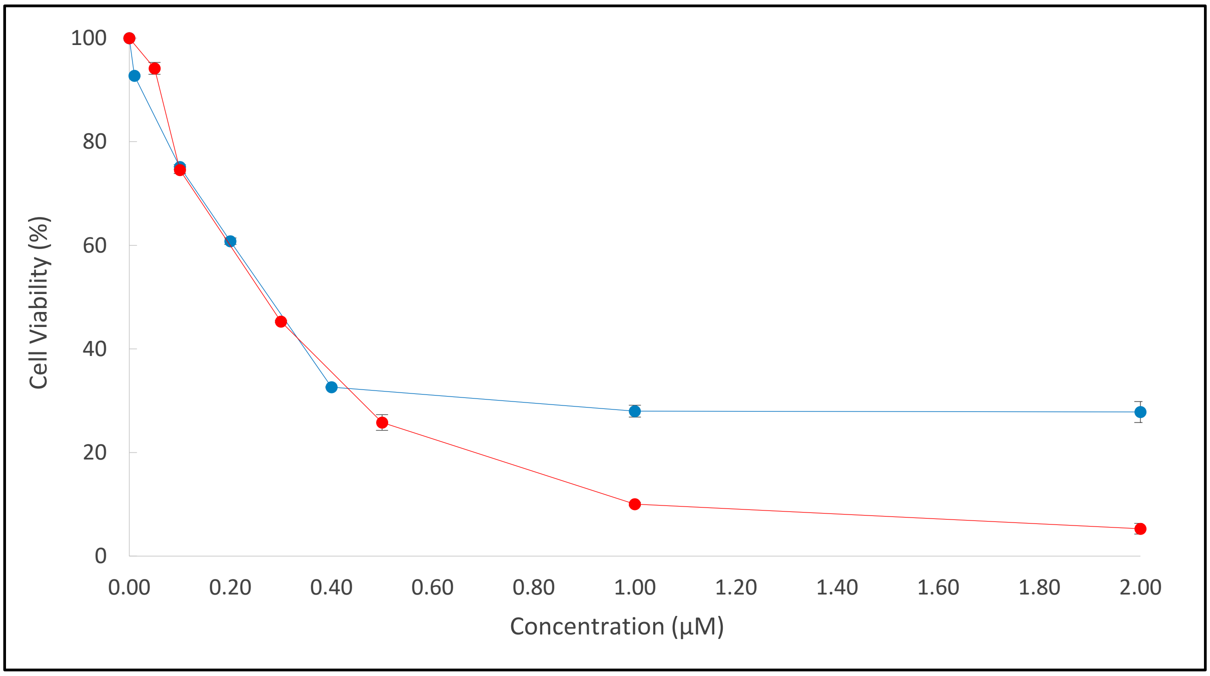

Figure S12. Viability of DU-145 (●) and MCF-7 (●) cell lines determined using MTT assay following treatment with for 3-phenyl-1-(pyrid-2-yl)benzo[*e*][1,2,4]triazin-7(1*H*)-one (**4a**) under aerobic conditions for 72 h at 37 °C. Each data point is the mean of at least two independent experiments. The lines shown are trend lines.

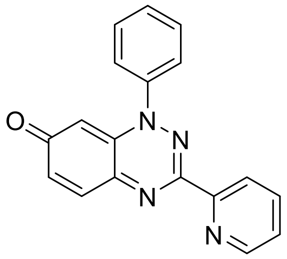

4b

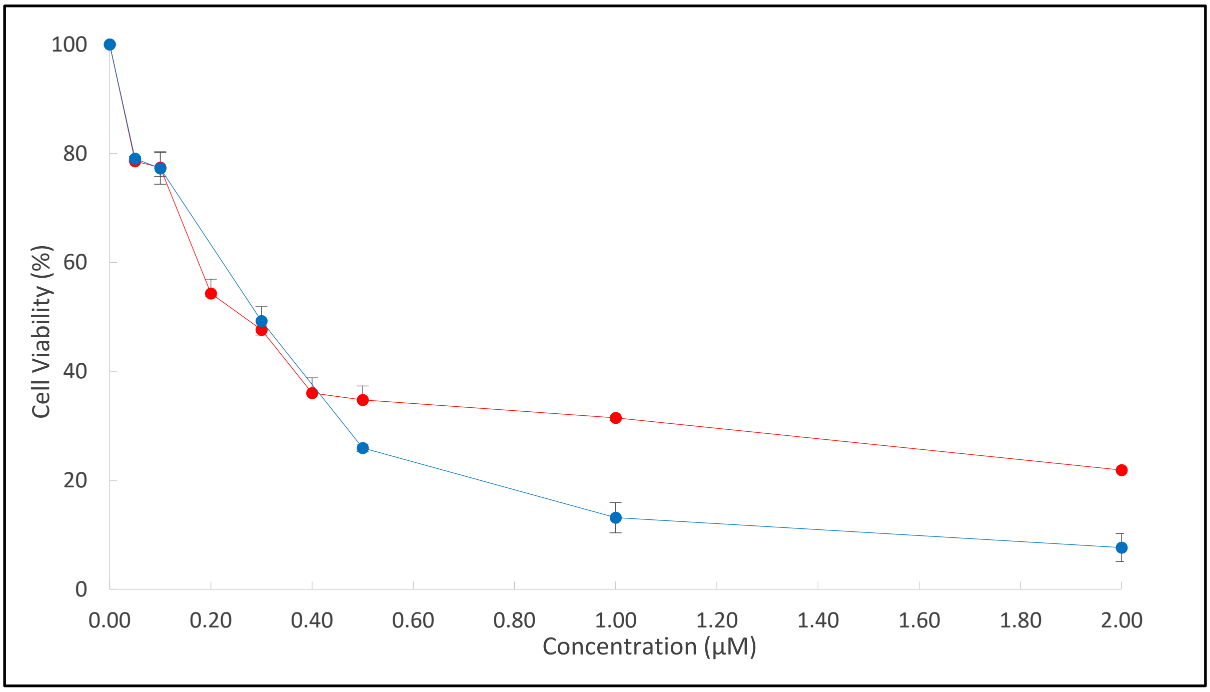

Figure S13. Viability of DU-145 (●) and MCF-7 (●) cell lines determined using MTT assay following treatment with 1-phenyl-3-(pyrid-2-yl)benzo[*e*][1,2,4]triazin-7(1*H*)-one (**4b**) under aerobic conditions for 72 h at 37 °C. Each data point is the mean of at least two independent experiments. The lines shown are trend lines.
